# Amygdalobacter indicium gen. nov., sp. nov., and Amygdalobacter nucleatus sp. nov., gen. nov.: novel bacteria from the family Oscillospiraceae isolated from the female genital tract

**DOI:** 10.1099/ijsem.0.006017

**Published:** 2023-10-03

**Authors:** Sujatha Srinivasan, Michele N. Austin, Tina L. Fiedler, Susan M. Strenk, Kathy J. Agnew, G. A. Nagana Gowda, Daniel Raftery, May A. Beamer, Sharon L. Achilles, Harold C. Wiesenfeld, David N. Fredricks, Sharon L. Hillier

**Affiliations:** 1 Vaccine and Infectious Disease Division, Fred Hutchinson Cancer Center, Seattle, WA, USA; 2 Magee-Womens Research Institute, Pittsburgh, PA, USA; 3 Division of Gynecologic Oncology, Department of Obstetrics & Gynecology, University of Washington Medical Center, Seattle, WA, USA; 4 Northwest Metabolomics Research Center and Mitochondrial and Metabolism Center, Anesthesiology and Pain Medicine, University of Washington Medical Center, Seattle, WA, USA; 5 Public Health Sciences Division, Fred Hutchinson Cancer Center, Seattle, WA, USA; 6 University of Pittsburgh School of Medicine, Department of Obstetrics, Gynecology and Reproductive Sciences, Pittsburgh PA, USA; 7 Department of Medicine, University of Washington, Seattle, WA, USA

**Keywords:** BVAB2, bacterial vaginosis, genital tract, human vagina, *Oscillospiraceae*

## Abstract

Four obligately anaerobic Gram-positive bacteria representing one novel genus and two novel species were isolated from the female genital tract. Both novel species, designated UPII 610-J^T^ and KA00274^T^, and an additional isolate of each species were characterized utilizing biochemical, genotypic and phylogenetic analyses. All strains were non-motile and non-spore forming, asaccharolytic, non-cellulolytic and indole-negative coccobacilli. Fatty acid methyl ester analysis for UPII 610-J^T^ and KA00274^T^ and additional isolates revealed C_16 : 0_, C_18 : 0_, C_18:1_ω9c and C_18:2_ω6,9c to be the major fatty acids for both species. UPII 610-J^T^ had a 16S rRNA gene sequence similarity of 99.4 % to an uncultured clone sequence (AY724740) designated as Bacterial Vaginosis Associated Bacterium 2 (BVAB2). KA00274^T^ had a 16S rRNA gene sequence similarity of 96.5 % to UPII 610-J^T^. Whole genomic DNA mol% G+C content was 42.2 and 39.3 % for UPII 610-JT and KA00274^T^, respectively. Phylogenetic analyses indicate these isolates represent a novel genus and two novel species within the *Oscillospiraceae* family. We propose the names *Amygdalobacter indicium* gen. nov., sp. nov., for UPII 610-J^T^ representing the type strain of this species (=DSM 112989^T^, =ATCC TSD-274^T^) and *Amygdalobacter nucleatus* gen. nov., sp. nov., for KA00274^T^ representing the type strain of this species (=DSM 112988^T^, =ATCC TSD-275^T^).

Bacteria within the order *Clostridiales* are a taxonomically diverse group of organisms comprised of many paraphyletic families. Since 1975, many groups have utilized genomic studies to reorganize and reclassify their phylogenetic relationships using rRNA homology [1,5]. Novel organisms have recently been described and assigned to the order, and in 2009, the family *Ruminococcaceae* was proposed to accommodate a morphologically, phenotypically, and physiologically diverse group of genera within rRNA clusters III and IV [6,7].

Technological advances in genotypic classification using protein and whole genomic sequencing have revealed further misclassification within these clusters. In 2018, Zhang *et al*. proposed reclassification of most members of clostridial cluster III into a novel family, *Hungateiclostridiaceae,* which are taxonomically distinct from the other *Ruminococcaceae* family members [8]. In a 2019 taxonomic note, Tindall communicated that while reclassification is most likely justified based on the rules of the International Code of Nomenclature of Prokaryotes, both *Hungateiclostridiaceae* and *Ruminococcaceae* are validly published yet illegitimate names, and members of both families must remain recombined under the prioritized family name *Oscillospiraceae* until further work and emendation of the family occurs [9]. While the reclassification and creation of new family names to encompass these genera are outside of the scope of this work, we characterized two distinct novel species and an additional isolate of each species (UPII 610-J^T^, CHIC02 1186E3-8) and (KA00274^T^, ACE 1-034E1-9) representing members of a novel genus within the family *Oscillospiraceae*. Using phenotypic and genotypic approaches to characterize these strains, we propose the names *Amygdalobacter indicium* gen. nov., sp. nov. for UPII 610-J^T^ and *Amygdalobacter nucleatus* gen. nov., sp. nov. for KA00274^T^.

In 2005, Fredricks *et al*. used cultivation-independent methods to characterize the vaginal microbiota of women with and without bacterial vaginosis (BV) [10]. Three novel organisms within the *Clostridiales* order were detected that are highly specific for BV, and the terms bacterial vaginosis-associated bacterium 1, 2 and 3 (BVAB1, BVAB2, and BVAB3) were used to designate these microorganisms [10]. Several investigators have reported that BVAB2 is associated with the acquisition, recurrence, and persistence of BV, as well as adverse genital tract sequelae in women and it has been detected in men evaluated for nongonococcal urethritis and infertility [11,32]. Because of its strong independent association with BV, BVAB2 has become a target in three of four commercially available point of care multiplex assays, as a sensitive and specific predictor for BV diagnosis in the clinical setting [33]. Molecular data have suggested that BVAB2 is associated with clue cells, another diagnostic feature used in the clinical diagnosis of BV [15,22].

To date, only one of three BVABs within the *Clostridiales* order, *Mageeibacillus indolicus* (formerly BVAB3), has been cultivated, characterized, and validly named [34,35]. A combination of cultivation, direct 16S rRNA gene sequencing and sequence comparison was used to isolate and characterize organisms from the female genital tract [34,36, 37]. This work describes BVAB2 (UPII 610-J^T^, CHIC02 1186E3-8) and another organism with 96.5 % 16S rRNA gene sequence similarity with BVAB2, termed here as BVAB2-like (KA00274^T^, ACE 1-034E1-9). While much research has been published regarding BVAB2 detection in the genital tract and adverse sequelae, the health impact of BVAB2-like bacterial colonization in the genital tract is unknown.

UPII 610-J^T^, CHIC02 1186E3-8 and ACE 1-034E1-9 were isolated using cultivation-based methods from endometrial biopsy samples as previously described [34]. UPII 610-J^T^ was isolated from a participant in a research study evaluating women for subclinical pelvic inflammatory disease (PID). CHIC02 1186E3-8 was isolated from a participant in a research study aimed at characterizing alterations in immune cell populations and genital tract microbiota after the initiation of contraception. ACE 1-034E1-9 was isolated from a participant in a randomized phase II clinical trial evaluating two outpatient treatment regimens for acute PID. All three studies were conducted in Pittsburgh, PA and protocols were approved by the University of Pittsburgh, Institutional Review Board (IRB approval numbers: PRO 010010112, STUDY19100126, PRO10010112, respectively).

KA00274^T^ was isolated from a vaginal swab sample as previously described [36,37]. That study, aimed at isolating and characterizing novel isolates from the human vagina, was conducted in Seattle, WA and approved by the Fred Hutchinson Cancer Center Institutional Review Board (IRB approval number: IR7363).

*Fastidiosipila sanguinis* CCUG 47711 was obtained from the Culture Collection University of Gothenburg, Sweden (CCUG) and used as a reference strain. This strain was chosen because it is within *Clostridium* rRNA cluster III of the *Oscillospiraceae* family, was isolated from humans and was characterized independently outside of our laboratory. Characteristics of *F. sanguinis* were obtained from testing performed in this study and the original manuscript [38].

All participants provided written informed consent prior to participation in study activities. All isolates of interest and *F. sanguinis* were plated on commercially prepared Brucella agar supplemented with 5 % laked sheep blood, hemin and Vitamin K (BRU) (Hardy Diagnostics, Santa Maria, CA) and incubated in anaerobic atmospheric conditions. Prior to testing and when testing was complete, isolates were confirmed for identity and purity using direct 16S rRNA gene sequencing and sequence comparison.

The capacity to grow in broth was assessed for all novel strains prior to additional biochemical testing. Strains were tested for growth in the following in-house prepared broths; chopped meat carbohydrate broth (CMC), peptone yeast glucose modified medium (PYG-mod), Brucella broth, Brucella broth modified, clostridial reinforced medium without the addition of agar and supplemented with 3 % fetal calf serum (CRM-FCS), a commercially prepared thioglycollate broth (Anaerobe Systems) and enriched anaerobe medium (EAM) with and without the addition of 3 % FCS and carbohydrates (4 g l^−1^ glucose, 1 g l^−1^ cellobiose, 1 g l^−1^ maltose, 1 g l^−1^ soluble starch). All isolates showed little to no turbidity in any of the broths tested after incubation; therefore, viability was confirmed by subculturing the broths to solid media. The most consistent viability for all isolates was found using CRM-FCS. Based on recommendations for enhancement of Gram-positive organisms by Summanen *et al*., thioglycollate and PYG mediums were supplemented with the separate addition of 5 % horse serum, 5 % lactate, 0.5 % arginine and 0.5 % Tween 80 if not included in the initial media composition [39] and supplementation did not stimulate growth. Given the inconsistent increase in turbidity in liquid growth media, biochemical testing was conducted using substrates on solid agar media. Media details and recipes for in-house broths can be found in Supplement A, available in the online version of this article.

Colony morphology was characterized at both 2 and 7 days growth using a dissecting microscope adjusted to 20×. Colony morphology for UPII 610-J^T^ and CHIC02 1186E3-8 following 2 days incubation revealed colonies that were too small to be characterized. Following 7 days incubation, examination revealed cream, circular, flat, entire, opaque, smooth and slightly shiny, punctiform colonies 0.3 mm in diameter. Following 2 days incubation, KA00274^T^ and ACE 1-034E1-9 produced colonies that were cream, circular, flat, entire, opaque, smooth and slightly shiny, punctiform colonies 0.2 mm in diameter. Seven days of incubation revealed a larger colony, 0.8 mm in diameter, with the development of a slight viscid colony that was difficult to remove from the agar surface and inoculating loop.

Primary examination of Gram-stain and cellular morphology was performed with a light microscopy at 1000× magnification. Cellular morphology for all novel strains were Gram-stain-negative, short pleomorphic, coccobacillary rods, with blunt or tapered ends and often almond-shaped. *F. sanguinis* cellular and colony morphology were consistent with the original description [38].

Initial biochemical testing included motility testing and demonstration of sporulation, aerotolerance, testing for catalase (3 and 30 %) (ThermoFisher Scientific, Waltham, MA), urease and oxidase (Becton Dickinson), spot indole (prepared in-house), susceptibility to the special-potency antimicrobial test discs, vancomycin (5 µg), kanamycin (1000 µg), and metronidazole (50 µg) (Hardy Diagnostics), colistin (10 µg) (Becton Dickinson) and bile disc (1 mg) (prepared in-house) [39,40]. Additional biochemical testing included: assessment for lipase and lecithinase activity on Egg Yolk agar (Becton Dickinson), hydrogen sulphide production in SIM media (Hardy Diagnostics) and fermentation of glucose, hydrolysis of DNA, starch and casein using the Anaerobic Gram-Positive ID Quad plate (Remel, Lenexa, KS). Incubation times for Egg Yolk agar, SIM media and the Quad plate were longer (7 days) than the manufacturer’s recommendations to allow for sufficient growth. All biochemical testing was performed in duplicate. Some biochemical testing platforms resulted in duplicate testing for some substrates and thus confirmed results. Table 1 provides a comparison of defining characteristics for the novel isolates, *F. sanguinis* and select members of *Oscillospiraceae*.

**Table 1.** Comparison of defining characteristics for selective related organisms within the *Oscillospiraceae* family

	1	2	3	4	5	6	7	8	9	10	11	12
% Similarity to BVAB2 (AY724740)	99.8	99.9	97.7	97.5	89.9	87.1	89.7	89.6	87.4	88.9	87.6	87.4
Defining Characteristics:												
Aerotolerance	OA	OA	OA	OA	OA	A/M	OA	OA	OA	OA	OA	OA
Cell morphology	CB	CB	CB	CB	Rod	Cocci	CB	Rod	Rod	Rod	Rod	Rod
Cell length (µm)	1.7	1.24	1.39	1.3	1.25	nd	1.2–1.8	2–3	3.1	2.5–5.0	3.5	2.5–8.0
Gram-stain	−	−	−	−	−	+	+	−	−	+/-	−	+
Motility	−	−	−	−	−	−	−	+	+	NO	+	−
Flagella	−	−	−	−	−	−	−	nd	PB	P	P	−
Sporulation	−	−	−	−	−	−	+	−	+	+	+	+
Presence of:												
Catalase	−	−	−	−	−	−	−	+	−	−	−	nd
Oxidase	−	−	−	−	−	−	−	+	nd	nd	−	nd
Urease	−	−	−	−	−	−	−	−	nd	nd	nd	nd
Lipase	−	−	−	−	−	−	−	nd	−	−	−	nd
Lecithinase	−	−	−	−	−	−	−	nd	−	−	−	nd
Nitrate reduction	−	−	−	−	−	−	−	−	−	−	−	nd
Cellulose degradation	−	−	−	−	−	−	−	−	+	+	+	−
												
Production of:												
Indole	−	−	−	−	+	−	nd	−	−	−	nd	nd
Hydrolysis of:												
Gelatin	−	−	−	−	−	−	−	−	−	−	nd	nd
Aesculin	−	−	−	−	−	−	+	nd	+	+	−	nd
												
Growth at 45 °C	−	−	−	−	−	−	−	−	+	+	−	+
Production of:												
Acetic acid	+	−	−	−	nd	+	+	+	+	+	+	+
Fumaric acid	+	+	+	+	nd	+	+	nd	nd	nd	nd	nd
Formic acid	+	+	+	+	nd	−	nd	nd	−	+	nd	nd
Lactic acid	−	−	−	−	nd	+	+	nd	+	+	nd	nd
Phenylacetic acid	+	−	+	+	nd	+	nd	nd	nd	nd	nd	nd
Propionic acid	+	+	+	+	nd	w	nd	nd	nd	nd	nd	nd
Pyruvic acid	+	+	+	+	nd	w	nd	nd	nd	nd	nd	nd

1-*Amygdalobacter indicium*, UPII 610-JT; 2*-A*. *indicium*, CHIC02 1186E3-8; 3-*Amygdalobacter nucleatus*, KA00274T; 4-*A*. *nucleatus*, ACE 1-034E1-9; 5-*Mageeibacillus indolicus* [37]; 6-*Fastidiosipila sanguinis* [38]; 7-*Saccharofermentans acetigenes* [67]; 8-*Ercella succinigenes* [68]; 9-*Thermoclostridium caenicola* [69]; 10-*Acetivibrio clariflavus* [69]; 11-*Ruminiclostridium sufflavum* [70]; 12-*Petroclostridium xylanilyticum* [8].

* Data for *F. sanguinis* was generated in our current study. Data for other organisms included for comparison was referenced from publications outside of this work. See references. Nitrate reduction was determined using API Rapid ID 32A.

-Negative+Positive+/-VariableCBCoccobacillaryndNot determinedNONot observedOAObligately anaerobicPPeritrichousPBPolar BundleWWeak

To assess for motility, cells were dispersed in CMC broth and visualized with light microscopy at 400×. All strains tested were non-motile. In addition, spores were not observed for any isolate during primary examination or with the method for inducing sporulation as described by Stevens, Bryant and Carroll [40]. Aerotolerance was tested for all strains by inoculation onto BRU agar and incubated separately in 6 % CO_2_ for 48 h or in an AnaeroPack System, with Pouch-MicroAero, microaerophilic gas generating sachet and jar (Mitsubishi Gas Chemical Company) at 37 °C for 7 days. All novel strains tested were obligately anaerobic. *F. sanguinis*, used as a control, was also able to grow in a microaerophilic atmosphere as previously described [38].

All novel strains and *F. sanguinis* were catalase-, oxidase- and urease-negative; did not ferment glucose, hydrolyse milk or starch; were negative for DNase, lecithinase and lipase activity; did not produce indole or hydrogen sulphide; were sensitive to the special potency antimicrobial discs vancomycin and kanamycin but were resistant to colistin. Novel strains exhibited sensitivity to bile and metronidazole while *F. sanguinis* was resistant to both bile and metronidazole.

Optimal temperature for growth was determined by inoculating all strains onto BRU agar and separately incubating them anaerobically for 7 days at the following range of temperatures: 4, 23, 30, 37–42, 45 and 60 °C. Growth for UPII 610-J^T^, CHIC02 1186E3-8 and KA00274^T^, ACE1-034E1-9 was observed between 30 and 42 °C with an optimal temperature of 37 °C. Growth for *F. sanguinis* was observed between 23 and 42 °C with an optimal temperature of 37 °C. pH testing was performed using CRM-FCS. UPII 610-J^T^ and CHIC02 1186E3-8 were viable after 1 day of incubation at pH 5.0–7.5. KA00274^T^ and ACE 1-034E1-9 were viable after 1 day of incubation at pH 5.5–7.5. pH greater than 7.5 was not tested due to the instability of the pH in the necessary anaerobic atmosphere required for growth. All isolates grew consistently on BRU, which has a pH of 7.0+/-0.3. Cell wall structure assessed by Gram-stain and light microscopy can be unreliable for obligately anaerobic organisms [41,42]. The string test method developed by Halebian *et al*. [43] was also performed to aid in Gram-stain evaluation and no strain was found to be positive. This result combined with the special-potency antimicrobial disc susceptibility profile (see Table 2), suggest that the novel species are Gram-positive organisms.

**Table 2.** Antimicrobial susceptibility testing for novel strains

Strain	ClindamycinS = <2 µg µl^−1^I=4 µg µl^−1^R = >8 µg µl^−1^	MetronidazoleS = <8 µg µl^−1^I=16 µg µl^−1^R = >32 µg µl^−1^	Tinidazole^*^	Secnidazole^*^
***Amygdalobacter indicium***				
UPII 610-J^T^	0.125 µg ml^−1^	0.5 µg ml^−1^	0.125 µg ml^−1^	0.5 µg ml^−1^
CHIC02 1186E3-8	0.03 µg ml^−1^	8 µg ml^−1^	4 µg ml^−1^	8 µg ml^−1^
***Amygdalobacter nucleatus***				
KA00274^T^	0.03 µg ml^−1^	8 µg ml^−1^	4 µg ml^−1^	16 µg ml^−1^
ACE 1-034E1-9	0.03 µg ml^−1^	16 µg ml^−1^	32 µg ml^−1^	32 µg ml^−1^

*Breakpoints of resistance are not listed for tinidazole and secnidazole. The lowest concentrations at which we noted a clearance zone are shown.

I, Intermediate; R, ResistantSSensitiveS, Sensitive

Cellulose degradation, an important defining characteristic of the *Oscillospiraceae* family, was assessed using commercially prepared modified Cellulose agar (Hardy Diagnostics) as well as a rapid method for the detection of bacterial cellulases using carboxymethylcellulose agar plates and Gram’s iodine developed by Kasana *et al.* [44]. Incubation time for both medias was extended to 7 days prior to screening for cellulase production. All novel strains and *F. sanguinis* were non-cellulolytic (Table 1).

API rapid ID 32A, API ZYM and API 20A (bioMérieux USA, Durham, NC) were completed for each strain; reactions to substrates for the ID 32A and ZYM panels can be found in Table 3. Substrate reactions for UPII 610-J^T^, CHIC02 1186E3-8 and KA00274^T^, ACE 1-034E1-9 were very similar. Only β-galactosidase differed between species and was consistently positive for both KA00274^T^ and ACE 1-034E1-9 and consistently negative for UPII 610-J^T^ and CHIC02 1186E3-8 in both the API rapid 32A and API ZYM panels (Table 3).

**Table 3.** Biochemical reactions to API substrates

	*A. indicium*		*A. nucleatus*		*F. sanguinis*
	UPII 610-J^T^	CHIC02 1186E3-8	KA000274^T^	ACE 1-034E1-9	CCUG 47711
**API Rapid 32A**					
Urease	−	−	−	−	−
Arginine DiHydrolase	−	−	−	−	−
α-Galactosidase	^+^	^+^	^+^	^+^	^+^
**β-Galactosidase**	−	−	^+^	^+^	−
β-Galactosidase-6-phosphate	−	−	−	−	−
α-Glucosidase	−	−	−	−	V
β-Glucosidase	−	−	−	−	V
α-Arabinosidase	−	−	−	−	−
β-Glucuronidase	−	−	−	−	V
N-acetyl-β-Glucosaminidase	V	^+^	^+^	V	^+^
Mannose fermentation	−	−	−	−	−
Raffinose fermentation	−	−	−	−	−
Glutamic acid decarboxylase	−	V	−	−	−
α-Fucosidase	−	−	−	−	^+^
Nitrate reduction	−	−	−	−	−
Indole production	−	−	−	−	−
Alkaline phosphatase	−	−	−	−	−
Arginine arylamidase	^+^	^+^	^+^	^+^	^+^
Proline arylamidase	^+^	^+^	^+^	^+^	^+^
Leucyl glycine arylamidase	^+^	^+^	^+^	^+^	^+^
Phenylalanine arylamidase	^+^	^+^	^+^	^+^	^+^
Leucine arylamidase	^+^	^+^	^+^	^+^	^+^
Pyroglutamic acid arylamidase	−	−	−	−	−
Tyrosine arylamidase	^+^	^+^	^+^	^+^	^+^
Alanine arylamidase	^+^	^+^	^+^	^+^	^+^
Glycine arylamidase	^+^	^+^	^+^	^+^	^+^
Histidine arylamidase	^+^	^+^	^+^	^+^	^+^
Glutamyl glutamic acid arylamidase	^+^	^+^	^+^	^+^	−
Serine arylamidase	^+^	^+^	^+^	^+^	^+^
**API ZYM**					
Alkaline phosphatase	−	−	−	−	−
Esterase	^+^	^+^	^+^	^+^	^+^
Esterase lipase	^+^	^+^	^+^	^+^	−
Lipase (Egg yolk)	V	V	−	V	−
Leucine arylamidase	^+^	^+^	^+^	^+^	^+^
Valine arylamidase	^+^	^+^	^+^	^+^	−
Cystine arylamidase	^+^	^+^	V	^+^	−
Trypsin	−	−	−	−	−
Alpha-chymotrypsin	^+^	^+^	^+^	^+^	−
Acid phosphatase	^+^	^+^	^+^	^+^	−
Naphthol-AS-BI-phosphohydrolase	^+^	^+^	^+^	^+^	−
α-Galactosidase	−	−	−	−	^+^
**β-Galactosidase**	−	−	^+^	^+^	−
β-Glucuronidase	−	−	−	−	^+^
α-Glucosidase	−	−	−	−	−
β-Glucosidase	−	−	−	−	−
N-acetyl-β-Glucosaminidase	−	−	−	−	^+^
α-Mannosidase	−	−	−	−	−
α-Fucosidase	−	−	−	−	−

Results listed for *F. sanguinis* were generated in this study.

+(+) Positive; (-) Negative; (V) Variable.

Substrate reactions listed for *F. sanguinis* in Table 3 were obtained during testing in our laboratory. Some variability in substrate results were observed between studies. To compare results generated in our study to those generated in Falsen *et al.*, variable reactions generated between studies were considered equivalent to either a positive or negative or variable result since all reactions were possible with repeat testing. Using this algorithm, we observed that substrate reactions for *F. sanguinis* in this study and previously published results were mostly consistent and only two substrate reactions, β-galactosidase and phenylalanine arylamidase, differed when comparing our results to the original study [38] (Table 3).

Carbohydrate fermentation was assessed using API 20A enzyme panels instead of pre-reduced anaerobically sterilized peptone-yeast medium (PRAS) with carbohydrate since strains could not be propagated in broth. All substrate reactions in the API 20A panel were negative (data not shown). Carbohydrate fermentation for *F. sanguinis* cannot be directly compared with our methods as Falsen *et al.* utilized PRAS media for testing [38]. Although two different methodologies were used, results confirm *F. sanguinis* is asaccharolytic.

Fatty acid methyl extraction (FAME) analysis [45] was conducted to determine cell wall composition (Table 4). Due to a necessity for anaerobic atmospheric conditions and poor growth in broth, standard growth conditions could not be used for propagation and analysis. Per guidance from the analysing laboratory, colonies for all strains were harvested directly from BRU agar at 7 days growth, resuspended heavily in sterile water and pelleted. Pellets were stored at −80 °C and shipped frozen on dry ice to Microbial ID (Newark, DE). Although proportions varied minimally, predominant fatty acids for *F. sanguinis* are similar to previously published results, confirming the adapted conditions used to prepare samples was sufficient to produce comparable results [38,46].

**Table 4.** Comparison of fatty acid methyl ester analysis (FAME) of novel strains with select organisms within the *Oscillospiraceae* family

Fatty acid composition	1	2	3	4	5	6	7	8	9	10	11	12
10 : 0	–	–	0.57	–	–	0.23	–	–	–	–	–	–
12 : 0	1.05	0.78	0.86	–	1.95	1.56	–	–	–	–	–	–
13 : 0 iso	–	–	–	–	–	–	–	–	1.36	–	–	–
13 : 0 anteiso	–	–	–	–	–	–	–	–	0.26	–	–	–
13 : 0 iso 3-OH	–	–	–	–	–	–	–	–	0.08	1.37	–	–
14 : 0	2.37	2.85	3.75	3.70	**11.09**	**10.56**	**7.4**	2.6	1.50	3.35	**7.7**	2.4
14 : 0 iso	–	–	–	–	–	–	3.2	**28.3**	1.99	0.94	**14.6**	1.0
14 : 0 anteiso	–	–	–	–	–	–	–	–	–	–	–	–
14 : 0 iso 3-OH	–	–	–	–	–	–	–	4.4	–	–	–	–
14 : 0 iso DMA	–	–	–	–	–	–	–	1.0	–	–	–	–
14 : 0 DMA	–	–	–	–	–	–	2.9	1.0	0.09	3.22	3.7	0.1
14 : 1 ω5c	–	–	–	–	–	0.38	–	–	–	–	–	–
15 : 0	1.11	1.08	1.08	1.24	3.05	1.86	0.9	–	–	0.62	–	–
15 : 0 iso	–	–	–	–	0.41	0.77	**27.5**	4.9	**18.85**	1.67	**16.1**	**34.7**
15 : 0 anteiso	–	–	–	–	0.66	0.93	**23.3**	2.0	**9.51**	–	**8.7**	**18.8**
15 : 0 iso 3-OH	–	–	–	–	–	–	–	0.4	–	–	–	–
15 : 0 iso DMA	–	–	–	–	–	–	**8.1**	2.8	0.58	1.74	**7.2**	1.9
16 : 0	**40.87**	**40.54**	**39.08**	**48.37**	**30.13**	**50.54**	**10.4**	2.7	**5.92**	**20.43**	4.8	**9.6**
16 : 0 iso	–	–	–	–	–	0.39	2.6	**5.9**	**22.97**	**23.70**	3.2	4.0
16 : 0 anteiso	–	–	–	–	–	–	–	1.5	–	–	–	–
16 : 0 iso 3-OH	–	–	–	–	–	–	–	–	–	–	–	–
16 : 0 iso DMA	–	–	–	–	–	–	0.6	**27.0**	–	–	–	–
16 : 0 iso AGE	–	–	–	–	–	–	–	–	–	–	–	–
16 : 0 DMA	–	–	–	–	0.93	–	2.6	**8.0**	0.29	**16.47**	**11.5**	2.9
16 : 0 AGE	–	–	–	–	–	–	–	–	–	–	–	–
16 : 0 3-OH	–	–	–	–	–	–	–	0.5	0.40	–	–	–
16 : 0 aldehyde	–	–	–	–	0.58	–	–	–	0.06	4.83	2.4	0.8
16 : 1	–	–	–	–	–	–	–	–	–	–	–	–
16 : 1 ω7c	1.69	1.78	1.48	1.65	1.41	2.40	–	–	1.18	–	–	–
17 : 0	1.24	1.19	1.35	–	1.56	–	–	–	–	–	–	–
17 : 0 iso	–	–	0.41	–	–	–	5.3	–	**8.18**	**5.42**	–	**10.2**
17 : 0 iso DMA	–	–	–	–	–	–	–	0.5	–	–	–	–
17 : 0 anteiso	0.52	0.39	0.55	–	0.49	0.77	2.0	–	**9.18**	1.22	–	**6.7**
17 : 0 anteiso 3-OH	–	–	–	–	–	–	–	–	1.31	–	–	–
17 : 0 anteiso DMA	–	–	–	–	–	–	–	–	0.21	–	–	–
18 : 0	**10.40**	**10.41**	**17.27**	**14.86**	**5.69**	**5.77**	2.1	0.4	0.24	0.73	–	1.2
18 : 0 iso	–	–	–	–	–	–	–	–	0.19	0.85	–	–
18 : 0 DMA	–	–	–	–	–	–	–	0.4	–	0.63	–	–
18 : 1	–	–	–	–	–	–	1.1	3.9	–	–	–	–
C18 : 1 ω6c	–	0.54	1.50	–	–	–	–	–	–	–	–	–
C18 : 1 ω9c	**34.07**	**31.52**	**23.11**	**23.53**	**31.52**	**16.32**	–	–	0.67	0.57	–	–
C18 : 2 ω6,9c	**6.68**	**8.40**	**7.38**	**6.65**	**7.62**	**6.24**	–	–	–	–	–	–
C18 : 2 ω6,9c/18 : 0 ANTE	–	–	–	–	–	**21.1**	–	–	–	–	–	–
19 : 0 Δ9,10 DMA	–	–	–	–	–	–	–	–	1.32	–	–	–
19 : 0 Δ11,12 DMA	–	–	–	–	–	–	–	–	0.22	–	–	–
Unknown 16.107	–	–	–	–	–	–	–	–	0.34	**7.87**	–	0.7
Unknown 17.103	–	–	–	–	–	–	–	–	0.15	1.84	–	1.6
Summed feature 1	–	–	–	–	–	–	–	–	0.30	1.70	–	–
Summed feature 3	–	–	–	–	–	–	–	–	4.69	–	–	0.5
Summed feature 8	–	0.52	0.41	–	1.04	0.46	–	–	–	–	–	–
Summed feature 10	–		1.19		1.86	–	–	–	–	–	–	–
Summed feature 13	–		–		–	–	–	–	0.51	0.20	–	0.7

1-*Amygdalobacter indicium* UPII 610-JT; 2*-A. indicium* CHIC02 1186E3-8; 3-*Amygdalobacter nucleatus* KA000274T; 4-*Amygdalobacter nucleatus* ACE 1-034E1-9; 5-*Mageeibacillus indolicus* [37]; 6-*Fastidiosipila sanguinis* [38]; 7-*Saccharofermentans acetigenes* [67]; 8-*Ercella succinigenes* [68]; 9-*Thermoclostridium caenicola* [69]; 10- *Acetivibrio clariflavus* [69]; 11-*Ruminiclostridium sufflavum* [70]; 12-*Petroclostridium xylanilyticum* [8].

* Summed feature 1 contained one or more of 13:1, 14:0 aldehyde and 11:0 2-OH; summed feature 3 contained 15:0 iso aldehyde and/or an unknown fatty acid; summed feature 8 contained 17:1ω8c and 17:2; summed feature 10 contained 18:1ω7c and an unknown fatty acid; summed feature 13 contained 15:0 anteiso DMA and/or 14:0 2-OH. Results for *F. sanguinis* were obtained from our study and not the orginal description. Values are percentages of total fatty acids. Fatty acids >5% are indicated in bold text.

--not detectedAGE, alkyl glycerol ethersDMADimethyl acetalDMA, Dimethyl acetal

Antimicrobial susceptibility was performed using the agar dilution method according to the Clinical Laboratory Standards Institute guidelines (CLSI) for anaerobic bacteria, ninth edition [47]. Susceptibilities to clindamycin, metronidazole, tinidazole and secnidazole were determined as these antibiotics are approved in the United States for BV treatment [48,49]. Breakpoints for anaerobes for clindamycin are <2 µg ml^−1^ (sensitive), 4 µg ml^−1^ (intermediate), >8 µg ml^−1^ (resistant) and <8 µg ml^−1^ (sensitive), 16 µg ml^−1^ (intermediate) and >32 µg ml^−1^ (resistant), for metronidazole, respectively. CLSI guidelines do not list breakpoints for tinidazole or secnidazole, so true susceptibility to these drugs cannot be determined [47,50]. Each novel strain and additional isolate were tested in triplicate and minimal inhibitory concentrations (MICs) are listed in Table 2. All novel strains tested were susceptible to clindamycin (Table 2). Most novel strains were also susceptible to metronidazole, except one strain (ACE 1-034E1-9) which exhibited intermediate susceptibility. Susceptibility of these novel strains to nitroimidazoles was unexpected as most non-spore forming anaerobic Gram-positive rods are typically resistant [51]. Further testing of additional strains is required to determine if ACE 1-034E1-9 is an outlier or if true variation in susceptibility exists among these bacteria.

Scanning electron microscopy (SEM; Fig. 1) and transmission electron microscopy (TEM; Fig. 2) were performed to further evaluate cellular morphology using methods described previously [37]. Bacterial cultures grown in CRM-FCS were centrifuged to form a pellet and fixed in 0.5 strength Karnovsky’s fixative (2.5 % glutaraldehyde, 2 % paraformaldehyde in 0.1 M sodium cacodylate buffer, pH 7.3) overnight at 4 °C for both SEM and TEM. Electron microscopy confirmed the pleomorphic coccobacillary shape visualized by Gram-stain. UPII 610-J^T^ cells are on average 1.7 µm in length and 0.75 µm in width and KA00274^T^ cells are on average 1.39 µm in length and 0.54 µm in width.

**Fig. 1.**
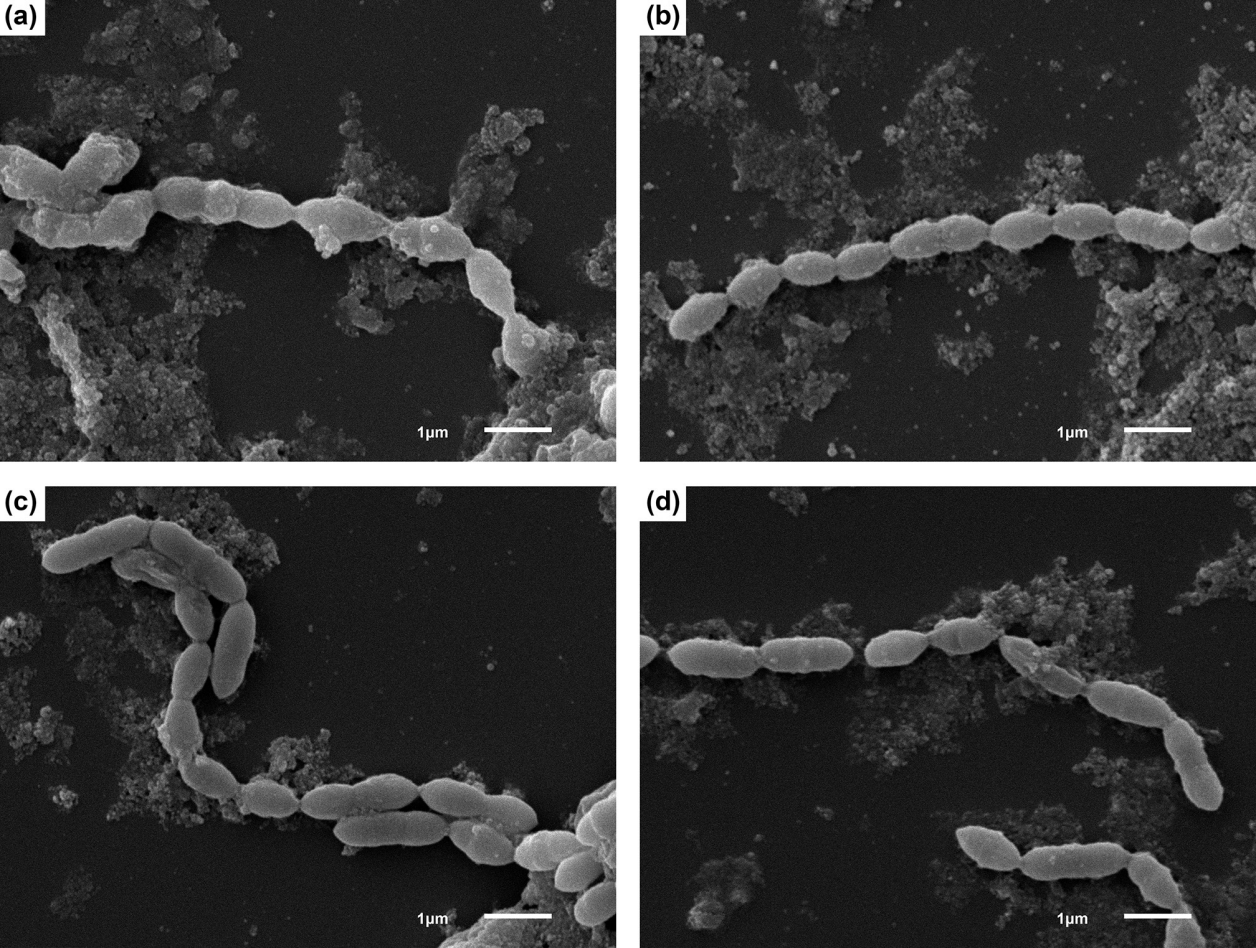
Scanning electron micrograph of cells of (**a**) *Amygdalobacter indicium* UPII 610-J^T^, (**b**) *A. indicium* CHIC02 1186E3-8, (**c**) *Amygdalobacter nucleatus* KA00274^T^ and (**d**) *A. nucleatus* ACE 1-034E1-9. Cells were cultured for 2 days in CRM-FCS. Bar, 1 µm.

**Fig. 2.**
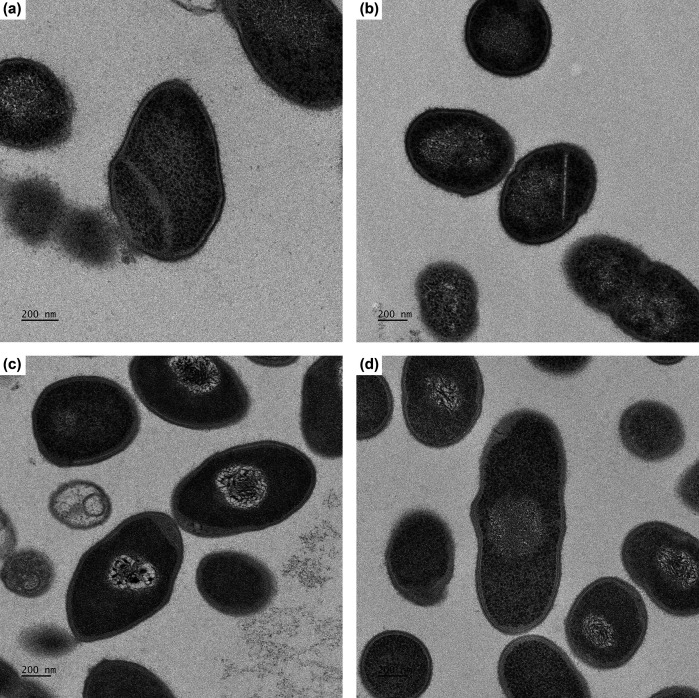
Transmission electron micrograph of cells of (**a**) *Amygdalobacter indicium* UPII 610-J^T^, (**b**) *A. indicium* CHIC02 1186E3-8, (**c**) *Amygdalobacter nucleatus* KA00274^T^ and (**d**) *A. nucleatus* ACE 1-034E1-9. Cells were cultured for 2 days in CRM-FCS. Bar, 200 nm.

To characterize the metabolic end products, *A. indicium, A. nucleatus, and F. sanguinis* cultures were grown in CRM-FCS for 48 h at 37 °C in duplicate. Cell supernatants were used for detection of short chain fatty acids and organic acids using ^1^H-NMR spectroscopy at the Northwest Metabolomics Research Centre at the University of Washington, as described previously [37]. Both UPII 610-J^T^ and KA00274^T^ produced fumarate, formate, phenylacetate, propionate, and pyruvate, and these acids were also detected in the second strains tested for each bacterial type except CHIC02 1186E3-8 which did not produce phenylacetate (Table 1, Fig. S1). UPII 610-J^T^ also produced acetate, similar to other members of the *Oscillospiraceae* compared in this study, but the second strain, CHIC02 1186E3-8, did not produce acetate. *F. sanguinis* produced lactate, but lactate production was not noted in the *Amygdalobacter* strains tested. Other end products that were not produced by *Amydalobacter* strains included butyrate, isobutyrate, 2-methylbutyrate, 2-aminobutyrate, succinate, valerate, and isovalerate (data not shown).

Bacterial cultures (20 to 40 ml) grown anaerobically for 48 h were subjected to DNA extraction using the MasterPure Gram Positive DNA purification kit (Epicentre, Madison WI) with several modifications to the manufacturer’s protocol [52]. Each pellet was resuspended in 300 µl TE buffer and briefly vortexed in a Lysing Matrix B bead tube (MP Biomedicals, Santa Ana CA). DNA was resuspended in 27 to 50 µl 0.1 X filtered (100 MWCO) Tris-EDTA buffer. Agarose gels (1%) were used to check that minimal shearing had occurred prior to sequencing. Single-molecule real-time sequencing (SMRT-Seq) was carried out on a Sequel IIe sequencer (Pacific Biosciences, Menlo Park, CA). Subread processing was conducted with SMRTlink v10.0.1. Raw CCL reads from the Sequel IIe instrument were transformed to CCS reads using the pbccs utility version 5.0.0. Quality control of the raw CCL reads was performed using SequelTools [53]. Assembly was performed with UniCycler v0.4.7 (https://github.com/rrwick/Unicycler/releases) and genomes were annotated using Prokka v1.12 [54]. CheckM v1.1.3 was used to assess genome completeness and quality [55]. BUSCO v5.2.2 was used to estimate contamination [56].

The genome sequences for all four isolates were deposited to NCBI (Accessions in Table 5). The 16S rRNA gene sequences for each isolate was obtained by sequencing the 16S rRNA gene as previously described [34] (Accessions in Fig. 3). All vaginal isolates characterized in this study each had two copies of the 16S rRNA gene. NCBI blast searches (https://blast.ncbi.nlm.nih.gov/Blast.cgi) [57] of the 16S rRNA gene sequences from UPII 610-J^T^ and KA00274^T^ resulted in matches to uncultivated clones from the human vagina. Importantly, UPII 610-J^T^ had a sequence identity of 99.9 % with the uncultured clone 123 f 23 (AY724740) designated as BVAB2 [10]. UPII 610-J^T^ and CHIC02 1186E3-8 had a sequence identity of 99.8 % indicating that they were bacterial strains within the same species. UPII 610-J^T^ had a sequence identity of 96.5 % to KA00274^T^ indicating that they were different species. KA00274^T^ and ACE 1-034E1-9 had a sequence identity of 99.7 % suggesting that they were two strains within the same species. A multiple sequence alignment of the 16S rRNA genes from the vaginal isolates and 80 validly named type strains from the family *Oscillospiraceae* was created using the ClustalW algorithm and evolutionary relationships were inferred by using the maximum-likelihood method based on the Tamura-Nei model [58] in MEGAX [59]. The closest neighbour of UPII 610-J^T^ was KA00274^T^ and both strains were phylogenetically distinct to the other type strains in the same clade including *Mageeibacillus indolicus*, *Saccharofermentans acetigenes*, *Ercella succinigenes*, and *F. sanguinis* (Fig. 3). Sequence identities of the 16S rRNA genes from UPII 610-J^T^ and KA00274^T^ were <90 % when compared with the 16S rRNA genes of other members of this clade suggesting that UPII 610-J^T^ and KA00274^T^ represented a novel genus within the family *Oscillospiraceae*. Similar results were obtained when the evolutionary relationships between the vaginal isolates and members of the *Oscillospiraceae* were evaluated using the neighbour-joining method (Fig. S2). It is possible that there may be further changes to the family *Oscillospiraceae* based on placement of validly named species such as *Ruminococcus* and *Acetivibrio* species which are not all present within the same cluster in the phylogenetic tree (Fig. 3). However, the novel *Amygdalobacter* species reported in this study are members of *Oscillospiraceae* as currently described and cluster with each other.

**Table 5.** Comparison of genome characteristics of genital tract *Amydalobacter* isolates with selected members of the *Oscillospiraceae* that are validly named Strains: 1-*Amygdalobacter indicium*, UPII 610-J^T^; 2-*A. indicium*, CHIC02 1186E3-8; 3-*Amygdalobacter nucleatus*, KA00274^T^; 4-*A. nucleatus*, ACE 1-034E1-9; 5-*Mageeibacillus indolicus* [37]; 6-*Fastidiosipila sanguinis* [38]; 7-*Saccharofermentans acetigenes* [67]; 8-*Ercella succinigenes* [68]; 9-*Thermoclostridium caenicola* [69]; 10-*Acetivibrio clariflavus* [69]; 11-*Ruminiclostridium sufflavum* [70]; 12-*Petroclostridium xylanilyticum* [8]. nd: genome data not data available Mol % G+C data was obtained from the genome information provided by the Bacterial and Viral Bioinformatics Resource Centre (BV-BRC) 3.25.0 (https://www.bv-brc.org/) [54]. When genome data was not available, the DNA G+C content was obtained from the original publications

Strains	GenBank accession	Genome length (Mb)	No. of protein coding genes	No. of tRNA genes	No. of rRNA genes	DNA G+C content (mol %)
1	CP118866	1.56	1309	45	6	42.2
2	CP118868	1.56	1321	45	6	42.2
3	JARFNM000000000	1.61	1496	44	6	39.3
4	CP118869	1.52	1373	44	6	39.0
5	CP001850	1.81	1579	45	4	44.2
6	CP027226	1.79	1526	42	2	33.7
7	nd	nd	nd	nd	nd	55.6
8	nd	nd	nd	nd	nd	37.6
9	FQZP00000000	2.89	2839	43	3	50.9
10	CP003065	4.90	4410	60	18	35.7
11	QKMR00000000	4.40	4020	52	6	38.7
12	NPML00000000	3.86	3945	63	16	37.2

**Fig. 3.**
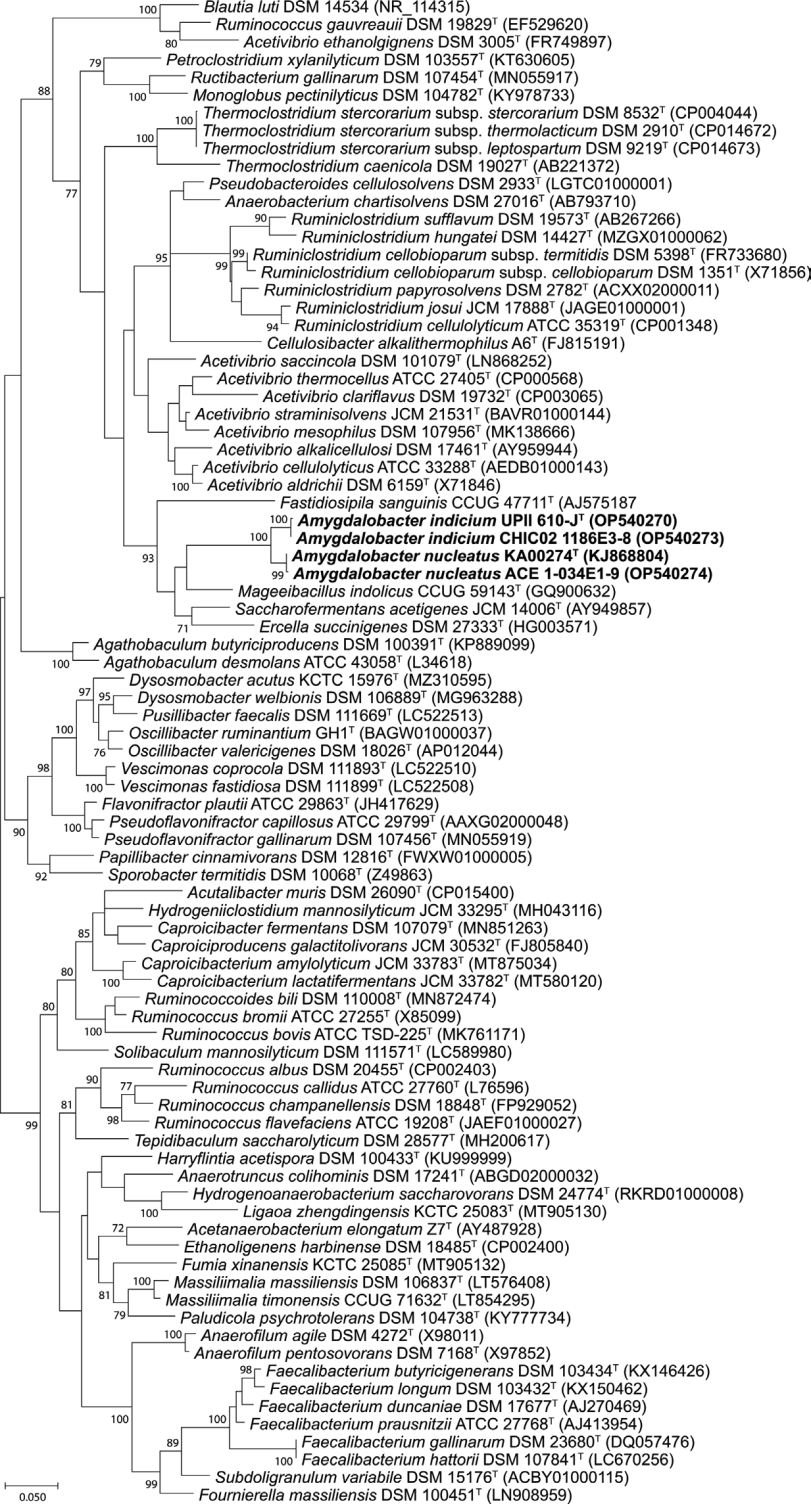
Molecular phylogenetic analysis by maximum likelihood method based on 16S rRNA gene sequences showing the phylogenetic positions of *Amygdalobacter indicium* UPII 610-J^T^, *A. indicium* CHIC02 1186E3-8, *Amygdalobacter nucleatus* KA00274^T^ and *A. nucleatus* ACE 1-034E1-9 in comparison with 80 validly named members of the family *Oscillospiraceae*. However, the analysis is not an exhaustive representation of all members of the *Oscillospiraceae* which encompasses members previously belonging to the *Ruminococcaceae* and *Hungateiclostridiaceae*. Bacteria including *Muriventricola aceti* DSM 108267, *Merdimmobilis hominis* KCTC 25350, *Thermocaproicibacter melissae* DSM 114174 and *Zongyangia hominis* KCTC 25132 among others were not included in this phylogenetic analysis. Bootstrap values (based on 1000 replications) greater than or equal to 70 % are shown as percentages at each node. Bar, 0.05 substitutions per nucleotide position. *Blautia luti* DSM 14534 (NR_114315) from the family *Lachnospiraceae* was added as an outgroup.

Information regarding DNA G+C content, genome lengths, predicted protein-coding genes and RNA genes were extracted from the Bacterial and Viral Bioinformatics Resource Centre (BV-BRC) 3.25.0 (https://www.bv-brc.org/) (Table 5) [60]. Digital DNA–DNA hybridization (dDDH) and average amino acid identity (AAI) was used to evaluate the relatedness of UPII 610-J^T^ and KA00274^T^ with each other, and to other reference genomes available from validly named species from the family *Oscillospiraceae* used for comparison in this study (Fig. 4). *S. acetigenes* and *E. succinigenes* did not have a published genome and hence we did not compare the vaginal strains with these two isolates. Genome-based species delineation was conducted using Type (Strain) Genome Server (TYGS) with the recommended settings for Formula two independent of genome length and robust against incomplete draft genomes (http://tygs.dsmz.de) [61,63]. AAI was calculated using a web-based tool (http://enve-omics.ce.gatech.edu.aai) [64]. dDDH values of UPII 610-J^T^ and KA00274^T^ were lower than ≤70 % and AAI values were <95 % when compared with each other, and other selected members of the *Oscillospiraceae* (Fig. 4). Both cutoffs are typically used for delineating a novel species [65,66]. In contrast, UPII 610-J^T^ and CHIC02 1186E3-8, and KA00274^T^ and ACE 1-034E1-9 each had dDDH values ≥70 % and AAI values >95 % suggesting that each pair represents two different strains within the same species respectively. The combined results for the phylogenetic, dDDH, and AAI analyses suggest that UPII 610-J^T^ and KA00274^T^ represent a novel genus within the *Oscillospiraceae* family, and the two type strains each represent a novel species within that genus.

**Fig. 4.**
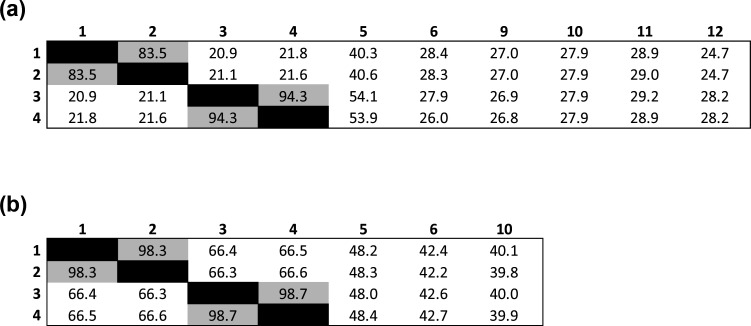
Genome-based species delineation using the (**a**) Genome Blast Distance Phylogeny approach. Values less than 70 % are indicative of a different species. Strains: 1-*Amygdalobacter indicium*, UPII 610-J^T^; 2*-A. indicium*, CHIC02 1186E3-8; 3-*Amygdalobacter nucleatus*, KA00274T; 4-*A. nucleatus*, ACE 1-034E1-9; 5-*Mageeibacillus indolicus* [34]; 6-*Fastidiosipila sanguinis* [38]; 7-*Saccharofermentans acetigenes* [62]; 8-*Ercella succinigenes* [63]; 9-*Thermoclostridium caenicola* [64]; 10-*Acetivibrio clariflavus* [64]; 11-*Ruminiclostridium sufflavum* [65]; 12-*Petroclostridium xylanilyticum* [8]. *S. acetigenes* and *E. succinigenes* did not have genomes available for this analysis. UPII 610-J^T^ and CHIC02 1186E3-8 belong to the same species with a dDDH value of 83.5 % (*A. indicium*). KA00274^T^ and ACE 1-034E1-9 belong to the same species with a dDDH value of 94.3 % (*A. nucleatus*). (**b**) Average amino acid identity (AAI) analyses. Values of <95 % indicate a novel species. UPII 610-J^T^ and CHIC02 1186E3-8 are the same species (98.3 % AAI) and KA00274^T^ and ACE 1-034E1-9 are the same species (98.7 % AAI).

## Description of *Amygdalobacter* gen. nov.

*Amygdalobacter*: (A.myg.da.lo.bac’ter. Gr. fem. n. *amygdale*, almond; N.L. masc. n. *bacter*, rod; N.L. masc. n. *Amygdalobacter*, an almond-shaped rod). Cells are Gram-positive, obligately anaerobic, mesophilic, non-motile, non-spore forming, asaccharolytic, non-cellulolytic. Predominant cell wall fatty acid methyl esters are unbranched saturated and unsaturated forms. The type species is *Amygdalobacter indicium*.

## Description of *Amygdalobacter indicium* sp. nov.

*Amygdalobacter indicium* (in.di’cium. L. neut. n. *indicium*, a sign, pertaining to its strong independent association with bacterial vaginosis when present in the female genital tract).

Colonies are cream, circular, flat, entire, opaque, smooth, slightly shiny, punctiform, and are 0.3 mm in diameter following 7 days anaerobic incubation at 37 °C on Brucella agar supplemented with 5 % laked sheep blood, hemin and Vitamin K. Cells are non-motile, non-spore forming, Gram-positive coccobacilli but stain Gram-negative, are approximately 1 µm in length and 600 nm in width and occur in chains. Strains are sensitive to vancomycin, kanamycin, and bile but resistant to colistin; negative for catalase, oxidase, urease, lipase and lecithinase activity, nitrate reduction and cellulose degradation; does not produce indole, hydrogen sulphide or hydrolyse gelatin, aesculin, starch, DNA or milk. Optimal temperature for growth is 37 °C. Asaccharolytic, but enzymatic activities are present for: α-galactosidase, N-acetyl-β-glucosaminidase, arginine arylamidase, proline arylamidase, leucyl glycine arylamidase, phenylalanine arylamidase, leucine arylamidase, tyrosine arylamidase, alanine arylamidase, glycine arylamidase, histidine arylamidase, glutamyl glutamic acid arylamidase, serine arylamidase, valine arylamidase, cysteine arylamidase, esterase, esterase lipase, alpha-chymotrypsin, acid phosphatase and naphthol-AS-BI-phosphohydrolase. Variable activity was present for glutamic acid decarboxylase and lipase. Major metabolic end products produced are acetate, fumarate, formate, phenylacetate, propionate, and pyruvate. Susceptible to clindamycin and metronidazole. Predominant cell wall fatty acids are C_16 : 0_, C_18 : 0_, C_18:1_ω9c and C_18:2_ω6,9c.

The type strain is UPII 610-J^T^ (=DSM 112989^T^=ATCC TSD-274^T^), which was isolated from an endometrial biopsy sample from a woman being evaluated for PID. The DNA G+C content is 42.2 mol%. GenBank accession numbers of the 16S rRNA gene sequence and the whole genome sequence are OP540270 and CP118866 respectively.

## Description of *Amygdalobacter nucleatus* sp. nov.

*Amygdalobacter nucleatus* (nu.cle.a’tus. N.L. masc. adj. *nucleatus*, containing a nucleus, pertaining to the nucleoid visible on transmission electron microscopy).

Colonies are cream, circular, flat, entire, opaque, smooth, slightly shiny, punctiform, and are 0.8 mm in diameter following 7 days anaerobic incubation at 37 °C on Brucella agar supplemented with 5 % laked sheep blood, hemin and Vitamin K. Cells are non-motile, non-spore forming, Gram-positive coccobacilli but stain Gram-negative, are approximately 1.1 µm in length and 700 nm in width and occur in chains. Strains are sensitive to vancomycin, kanamycin, and bile but resistant to colistin; negative for catalase, oxidase, urease, lipase and lecithinase activity, nitrate reduction and cellulose degradation; does not produce indole, hydrogen sulphide or hydrolyse gelatin, aesculin, starch, DNA or milk. Optimal temperature for growth is 37 °C. Asaccharolytic, but enzymatic activities are present for: α-galactosidase, β-galactosidase, N-acetyl-β-glucosaminidase, arginine arylamidase, proline arylamidase, leucyl glycine arylamidase, phenylalanine arylamidase, leucine arylamidase, tyrosine arylamidase, alanine arylamidase, glycine arylamidase, histidine arylamidase, glutamyl glutamic acid arylamidase, serine arylamidase, valine arylamidase, cysteine arylamidase, esterase, esterase lipase, alpha-chymotrypsin, acid phosphatase and naphthol-AS-BI-phosphohydrolase. Variable activity was present for lipase. Major metabolic end products produced are fumarate, formate, phenylacetate, propionate, and pyruvate. Susceptible to clindamycin and metronidazole. Predominant cell wall fatty acids are C_16 : 0_, C_18 : 0_, C_18:1_ω9c and C_18:2_ω6,9c.

The type strain is KA00274^T^ (=DSM 112988^T^=ATCC TSD-275^T^), which was isolated from human vaginal fluid obtained from a woman with BV. The DNA G+C content of the type strain is 39.3 mol%. GenBank accession numbers of the 16S rRNA gene sequence and the whole genome sequence are KJ868804 and JARFNM000000000 respectively.

## supplementary material

10.1099/ijsem.0.006017 Uncited Supplementary Material 1.

## Abbreviations

AAI: average amino acid identity
BRU: brucella agar supplemented with 5% laked sheep blood, hemin and vitamin K
BV: bacterial vaginosis
BVAB2: bacterial vaginosis associated bacterium 2
BV-BRC: Bacterial and Viral Bioinformatics Resource Center
CCUG: Culture Collection, University of Gothenburg
CLSI: Clinical Laboratory Standards Institute guidelines
CMC: chopped meat carbohydrate broth
CRM-FCS: clostridial reinforced medium with 3% fetal calf serum
dDDH: digital DNA-DNA hybridization
EAM: enriched anaerobe medium
FAME: fatty acid methyl extraction
FCS: fetal calf serum
PID: pelvic inflammatory disease
PRAS: pre-reduced anaerobically sterilized peptone-yeast medium
PYG-mod: peptone yeast glucose modified medium
SMRT-Seq: single-molecule-real-time sequencing
TYGS: type (strain) genome server
