# Supplementary material for: Amygdalobacter indicium gen. nov., sp. nov., and Amygdalobacter nucleatus sp. nov., gen. nov.: novel bacteria from the family Oscillospiraceae isolated from the female genital tract
Source: Int J Syst Evol Microbiol. 2023 Oct 3;73(10):006017. doi: 10.1099/ijsem.0.006017 (PMC11318147; doi:10.1099/ijsem.0.006017)

SUPPLEMENT A

Media and Materials for Propagating Bacterial Strains

Medium or Materials	Source	Purpose
Litmus Milk	Becton Dickinson, Franklin Lakes, NJ	Freezing stocks in -80ºC
10% glycerol v/v	In-house	Freezing stocks in -80ºC
Brucella Agar supplemented with 5% Laked Sheep Blood, Hemin and Vitamin K	Hardy Diagnostics, Santa Maria, CA	Growth on plates, incubated in anaerobic atmospheric conditions
Thioglycollate Broth	Anaerobe Systems	Assessing sporulation
Gas Mixture, 90% N_2, 5% H_2, 5% CO_2	Matheson Tri-Gas, Montgomeryville, PA	Creating anaerobic atmospheric conditions
AS-580 anaerobic chamber	Anaerobe Systems, Morgan Hill, CA	Creating anaerobic atmospheric conditions
AnaeroPack® Jar System	Mitsubishi Gas Chemical Company, Tokyo, Japan	Creating anaerobic or microaerophilic atmospheric conditions
Anaerobic or Microaerophilic Atmosphere Generating Sachet	Mitsubishi Gas Chemical Company, Tokyo, Japan	Creating anaerobic or microaerophilic atmospheric conditions

SUPPLEMENT A

Chopped Meat Carbohydrate Broth (CMC)

Ingredients	Grams/Liter
Tryptone	30.0
Yeast extract	5.0
Dibasic potassium phosphate	5.0
L-cysteine (see below)	5.0
Hemin solution (see below)	10 mL
Vitamin K solution (see below)	0.2 mL
Dextrose	4.0
Cellobiose	1.0
Maltose	1.0
Starch	1.0
Cooked meat medium	0.4 gm/tube

Hemin solution: Dissolve 50 mg hemin in 1 mL of 1N NaOH. Add distilled water to bring up to 100 mL. Sterilize. Cool. Store at 2-8 ºC.

Vitamin K solution: Dissolve 0.1 mL vitamin K in 20 mL 95% ethanol. Filter-sterilize and store away from light at 2-8 ºC.

1. Combine all ingredients except cooked meat granules.
2. Dispense cooked meat granules in sterile anaerobic transport tubes with caps.
3. Add 8 mL of CMC broth to each tube containing cooked meat granules.
4. Autoclave, cool.
5. Reduce and tighten caps.

Peptone Yeast Glucose Broth (modified)

Ingredients	Grams/Liter
Distilled water	950 mL
Tryptone peptone	5.0
Peptone	5.0
Yeast extract	10.0
Beef extract	5.0
Dextrose	5.0
Dibasic potassium phosphate	2.0
Tween 80	1 mL
Salt solution (see below)	40 mL
Hemin solution	10 mL
Vitamin K solution	0.2mL
L-cysteine	0.5

Salts Solution

Ingredients	Grams/Liter
Calcium chloride dihydrate	0.25
Magnesium sulfate heptahydrate	0.5
Dibasic potassium phosphate	1.0
Potassium dihydrogen phosphate	1.0
Sodium bicarbonate	10.0
Sodium chloride	2.0

1. Combine ingredients.
2. Dispense 7 mL in sterile anaerobic transport tubes with caps.
3. Autoclave. Cool.
4. Reduce and tighten caps.

Clostridial Reinforced Medium with Yeast and Glucose without agar (CRM-YG)

Ingredients	Grams/Liter
Peptone	10.0
Beef extract	10.0
Yeast extract	8.0
Dextrose	15.0
Sodium chloride	5.0
Soluble starch	1.0
L-cysteine	0.5
Sodium acetate	3.0
Vitamin K	200 µL

1. Combine all ingredients except Vitamin K to distilled water and bring to a boil to dissolve all powders.
2. Adjust pH to 6.8 +/- 0.2. Add Vitamin K and mix.
3. Dispense 5 mL in anaerobic transport tubes with caps
4. Autoclave. Cool.
5. Reduce and tighten caps.

6. Immediately prior to inoculation of tube with organism, aseptically add 3% sterile fetal calf serum.

Brucella Broth Modified

Ingredients	Grams/Liter
BD BBL Brucella Broth powder	28.0
DL-Lactic acid	5.0
Casamino acids	1.5
L-arginine	0.1
Glycine	0.1
L-cysteine	0.1
L-tryptophan	0.1
Vitamin K solution	0.2 mL
Hemin solution	10 mL

1. Combine all ingredients.
2. Adjust pH to 5.8
3. Dispense 5 mL in anaerobic transport tubes with caps.
4. Autoclave. Cool.
5. Reduce and tighten caps.

ATCC medium 2210: Enriched Anaerobe Medium[a]

Ingredients	Grams/L
Trypticase peptone	30.0
Beef Extract	20.0

Yeast Extract	5.0
Dipotassium phosphate	5.0
0.025% Resazurin	4 mL
L-cysteine	0.5
Vitamin K	200 µL
Hemin	10 mL

1. Combine all ingredients except L-cysteine, vitamin K and hemin.
2. While stirring, bring to boil to dissolve.
3. Cool to room temperature and add L-cysteine, vitamin K and hemin.
4. Adjust pH to 7.0 +/- (0.2).
5. Autoclave.

[a]**American Type Culture Collection**.

file:///C:/Users/austinmn/Downloads/ATCC%20Medium%202210%20(1).pdf Accessed for reference7/7/2021.

Enriched Anaerobe Medium with Carbohydrates

Formula	Grams/L
Trypticase peptone	30.0
Beef Extract	20.0
Yeast Extract	5.0
Dipotassium phosphate	5.0
D-glucose	4.0
Cellobiose	1.0

Maltose monohydrate	1.0
Soluble starch	1.0
0.025% Resazurin	4 mL
L-cysteine	0.5
Vitamin K	200 µL
Hemin	10 mL

1. Combine all ingredients except L-cysteine, vitamin K and hemin.

2. While stirring, bring to boil to dissolve.

3. Cool to room temperature and add L-cysteine, vitamin K and hemin.

4. Adjust pH to 7.0 +/- (0.2).

5. Autoclave.

SUPPLEMENTARY FIGURE LEGENDS

Supplementary Fig. 1. Concentrations of short chain fatty acids produced after growth of bacterial cells in CRM-FCS for 2 days at 37ºC. Results reported are mean values of duplicate measurements. Concentrations (µM) are shown in the y-axis. Strains: 1-*Amygdalobacter indicium*, UPII 610-J^T; 2-*A. indicium*, CHIC02 1186E3-8; 3-*Amygdalobacter nucleatus*, KA00274T; 4-*A. nucleatus*, ACE 1-034E1-9; 5-*Fastidiosipila sanguinis* [38].

Supplementary Fig. 2. Molecular phylogenetic analysis by neighbor joining method based on 16S rRNA gene sequences showing the phylogenetic positions of *Amygdalobacter indicium* UPII 610-J^T, *A. indicium* CHIC02 1186E3-8, *Amygdalobacter nucleatus* KA00274^T and *A. nucleatus* ACE 1-034E1-9 in comparison with members of the family *Oscillospiraceae*. Bootstrap values (based on 1000 replications) greater than or equal to 70% are shown as percentages at each node. Bar, 0.02 substitutions per nucleotide position. *Blautia luti* DSM 14534 (NR_114315) from the family *Lachnospiraceae* was added as an outgroup.

Supplementary Figure 1

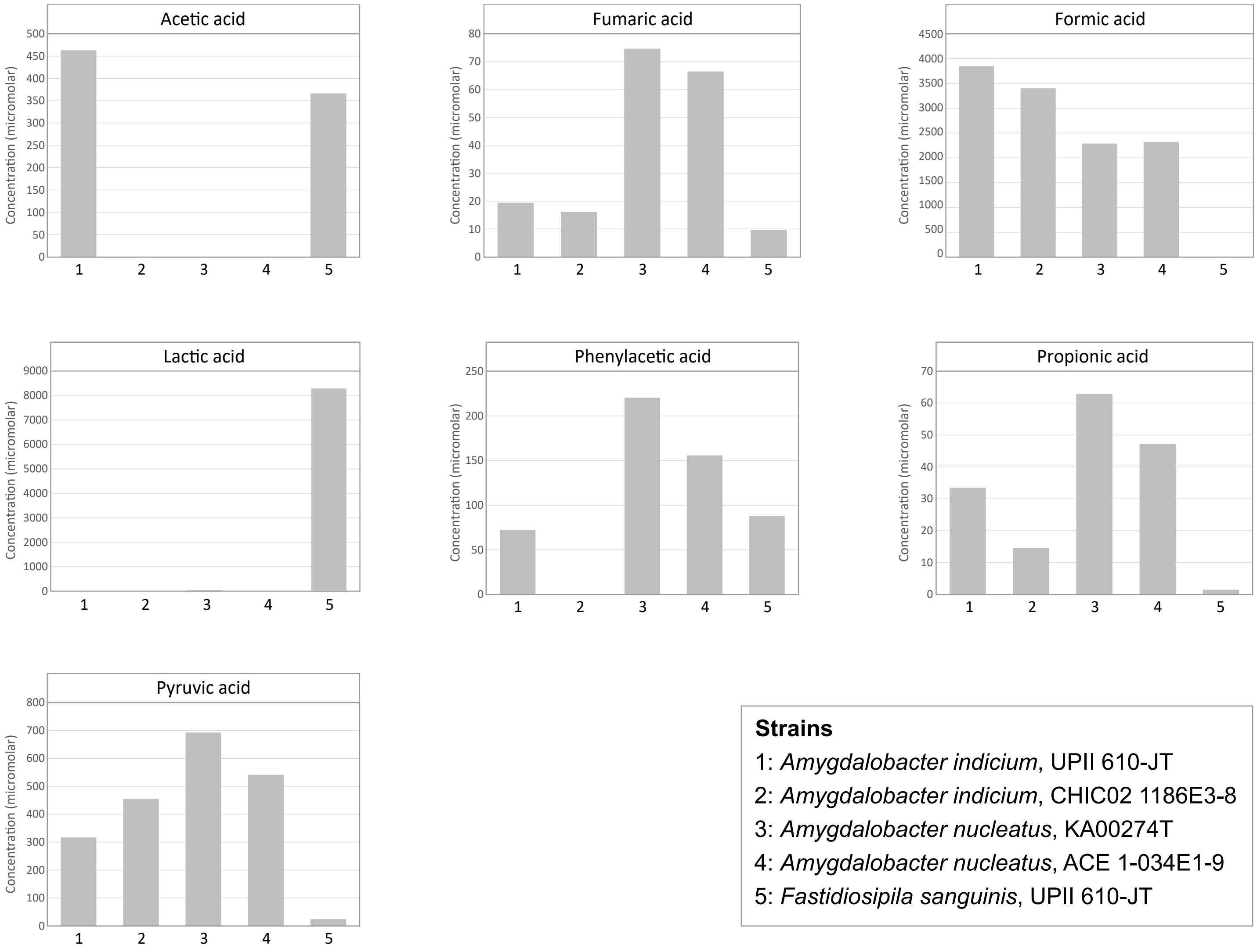

Supplementary Figure 2

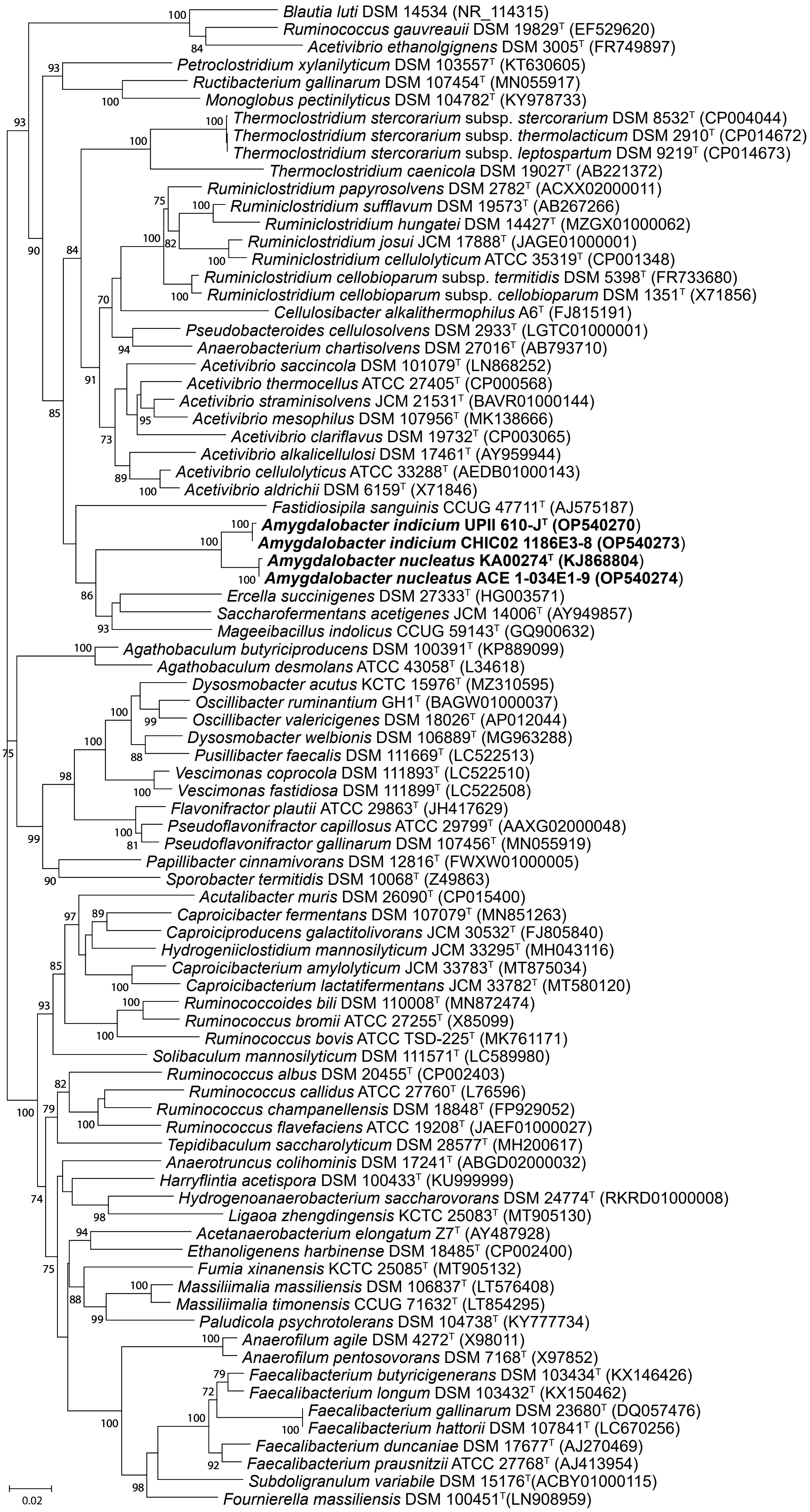

Supplement: Uncited Supplementary Material 1. [file ijsem-73-06017-s001.pdf]
